# The Current State of Osteoarthritis Treatment Options Using Stem Cells for Regenerative Therapy: A Review

**DOI:** 10.3390/ijms24108925

**Published:** 2023-05-18

**Authors:** Michael Thoene, Ewa Bejer-Olenska, Joanna Wojtkiewicz

**Affiliations:** 1 Department of Medical Biology, School of Public Health, University of Warmia and Mazury in Olsztyn, 10-561 Olsztyn, Poland; 2 Department of Pathophysiology, School of Medicine, University of Warmia and Mazury in Olsztyn, 10-082 Olsztyn, Poland

**Keywords:** mesenchymal stem cells, arthritis, tissue regeneration, canine animal models

## Abstract

Articular cartilage has very low metabolic activity. While minor injuries may be spontaneously repaired within the joint by chondrocytes, there is very little chance of a severely impaired joint regenerating itself when damaged. Therefore, any significant joint injury has little chance of spontaneously healing without some type of therapy. This article is a review that will examine the causes of osteoarthritis, both acute and chronic, and how it may be treated using traditional methods as well as with the latest stem cell technology. The latest regenerative therapy is discussed, including the use and potential risks of mesenchymal stem cells for tissue regeneration and implantation. Applications are then discussed for the treatment of OA in humans after using canine animal models. Since the most successful research models of OA were dogs, the first applications for treatment were veterinary. However, the treatment options have now advanced to the point where patients suffering from osteoarthritis may be treated with this technology. A survey of the literature was performed in order to determine the current state of stem cell technology being used in the treatment of osteoarthritis. Then, the stem cell technology was compared with traditional treatment options.

## 1. Introduction to Articular Cartilage

**Figure 1:**
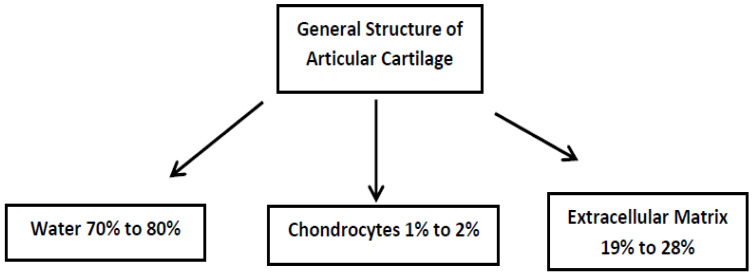
The general overall composition of adult articular cartilage. The majority of this cartilage is composed of water bonded to proteoglycans and extracellular matrix. Only chondrocytes can repair damaged cartilage. However, their low numbers and difficulty in migrating to where they are needed do not allow articular cartilage to be easily regenerated.

Articular cartilage is a typical hyaline cartilage. It consists primarily of chondrocytes and extracellular matrix including mostly type II collagen, a slight amount of collagen type VI, IX, XI, and XIV, as well as proteoglycans that bind water. In fact, approximately 70–80% of hyaline cartilage is composed of water. This tissue is characterized by a lack of direct innervation, nutrient blood supply, and lymphatic drainage. Its metabolic activity is low, and the proliferation of chondrocytes is very slow. These characteristics lie behind the poor self-healing processes and capacity for spontaneous repair. Cartilage injury without regenerative treatment is the reason that it affects the surrounding tissues, which leads to degeneration and osteoarthritis (OA) development [1,2,3]. Chondrocytes are trapped in the niches and cannot migrate to the damaged areas. In both the normal and pathological states, the environment of chondrocytes in the articular cartilage is very low in oxygen and tension, meaning that the chondrocytes are not under the same physical stresses as the type II collagen fibers, but they are also farther away from the fibers that may need to be repaired. All these tissue-specific environmental conditions create problems for regeneration [4]. The absence of cartilage vascularity does not allow progenitor cells to enter the cartilage, which could participate in and support the regenerative process [5,6]. In adult articular cartilage, cellular components are mostly postmitotic, with a low turnover rate, and have very limited self-repair abilities. The supply of glucose and oxygen to the cells mostly depends on diffusion from the synovial fluid and from the subchondral bone [7]. Figure 1 depicts the general overall composition of articular cartilage in adults and shows the small percentage of chondrocytes normally available to repair damaged tissues. 

**Figure 2:**
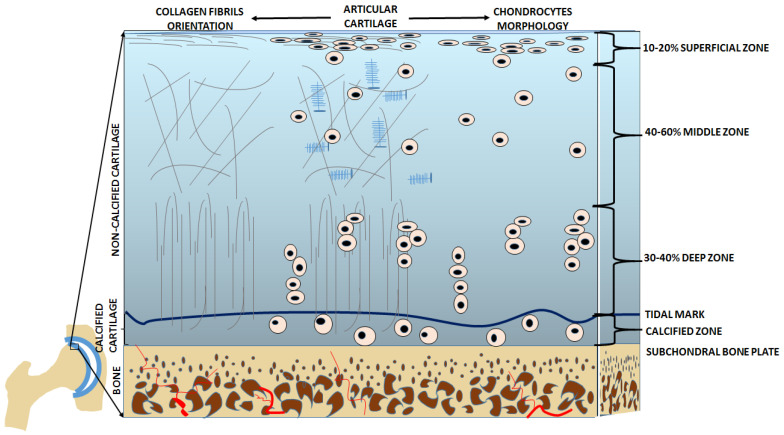
Healthy articular cartilage in cross-section. The right side of the image shows the organization of the chondrocytes within the various zones; while the left side of the image shows the structure of the collagen fibers within those same zones. Source: Own data.

In the articular cartilage structure, there is an articular surface acting as the outermost layer followed by four main zones. They are distinguished based on the shape of the chondrocytes, the composition of the extracellular matrix, and the orientation of the type II collagen fibers. The thinnest layer is the superficial or tangential zone (10–20%), which protects deeper layers from potential damage caused by articulation and is in direct contact with synovial fluid. Often abbreviated as the STZ, the superficial zone is aligned parallel to the articular surface, is tightly packed, and is composed mainly of Type II and IX collagen. Chondrocytes in the STZ are relatively flat. Directly under the STZ is an intermediate or middle zone, which is the thickest layer (40–60%). The function of this layer is to resist moderate compression. Therefore, it has thick fibrils of collagen that are neither perpendicular nor parallel, but are slanted, and it also contains water bonded to proteoglycans, which help to resist compressive forces. The chondrocytes in the middle zone are characterized by their low density and spherical shape. Directly under the middle zone is the deep or basal zone (30–40%), which has the highest resistance to compression. This resistance to compression comes from radially arranged thick collagen fibrils that are arranged perpendicularly to the articular surface. This zone has many proteoglycans but very little water. The chondrocytes in this layer are arranged parallel to the collagen fibers and are columnar. Under the deep zone is a tidal mark that separates it from the calcified zone. The main job of the calcified zone is to attach the cartilage to the bone. In this layer, there are very few chondrocytes and the few that reside there are hypertrophic [4,8]. Figure 2 illustrates the structure of healthy articular cartilage as described above, and Table 1 summarizes the characteristics of the chondrocytes and collagen fibers, along with the main functions of each zone. 

The chondrocytes are considered to be the only cellular component of the articular cartilage. Their main physiological function is the synthesis and degeneration of the extracellular matrix [9]. However, even adult articular cartilage contains MSCs and/or mesenchymal progenitor cells capable of differentiating into chondrocytes [7,10]. The highest concentration of stem cells is within the superficial zone of articular cartilage [8,11]. The chondrocytes of the articular cartilage are under physioxic/hypoxic conditions, but still have their normal metabolism (the oxygen gradient ranges from 10% to 1%, from the superficial to the deepest layers, respectively), and produce type II collagen and aggrecan (a proteoglycan made of chondroitin sulfate and keratin sulfate chains which can retain significant amounts of water). These main components of the extracellular matrix provide flexibility, viscoelasticity, pressure absorbance, and distribution [7,12].

All the structural and mechanical properties of the articular cartilage are subordinated into two major functions. These are the smooth gliding of the articular surfaces, as well as the protection of subchondral bone from mechanical stress [1]. In order to keep homeostasis in the tissue, there is a required homeostatic balance between the lytic, tissue-damaging mediators (cytokines that trigger catabolism, free radicals, proteases, and prostaglandins), as well as the reparative substances and physiological inhibitors (growth factors, inhibitors of catabolic cytokines, and degenerative enzymes) [9]. Aging is the major factor affecting the ability of chondrocytes to maintain and restore articular cartilage. With aging, the number of chondrocytes decreases, they begin to deteriorate (senescence), and more factors that cause apoptosis can be found. This is the main reason for the increased risk of articular cartilage degeneration with age [4].

Chondral and osteochondral defects mostly do not heal themselves without intervention, which leads to progressive joint degeneration [13]. If the cartilage repair processes take place, they are usually weak and nonfunctional, due to the replacement of damaged cartilage by a fibrocartilage-like scar tissue [4,14]. The biomechanical properties of fibrocartilage are inferior in quality when compared with hyaline cartilage, and effective joint restoration is not possible [15]. When compared to hyaline cartilage, fibrocartilage contains more collagen and fewer proteoglycans. Moreover, type I collagen is mostly represented. This has a lower compressive strength, elasticity, and wear resistance than type II collagen, which is specific and characteristic for “normal” articular cartilage [16]. Events that meet cartilage regeneration requirements occur in embryos, but quickly wear off after birth. In adults, this type of regeneration has never been noticed, and only the cartilage repair process is possible [1,15].

## 2. Osteoarthritis

Osteoarthritis (OA) is a chronic degenerative joint disease. The articular cartilage damage could be induced by biomechanical, metabolic, biochemical, or genetic factors. Increased risk factors of OA are obesity, aging, direct joint injury (a strong single event or cumulative micro-traumatic events), and/or a genetic predisposition. OA is a complex disease (it encompasses the entire joint) that activates all aspects of the immune system response. Progression of the disease involves cartilage, subchondral bone, synovium, tendons, ligaments, muscles, and even neural tissues. There is no doubt that in the late stages, OA is a systemic disease [17,18]. Two major categories of OA can be distinguished in general: (1) mechanical OA—healthy articular cartilage undergoes excessive loads leading to degeneration, and (2) structural OA—articular cartilage is weak, showing some abnormalities that contribute to rapid degradation. Even minor cartilage defects often lead to osteoarthritis [7]. Secondary OA might be the result of previous tendon or ligament injury, joint instability due to intra-articular fracture, or wear and tear of the articular cartilage. OA is one of the most challenging joint diseases and has several phenotypes [1,2]. However, we generalized these phenotypes into two major types, with numerous sub-types. 

Any of the body joints can be affected by OA. The knee is one of the most OA-affected joints in humans [19,20]. The knee, hip, elbow, carpal, tarsal, and vertebral joints are the most commonly osteoarthritic in both humans and pets [21,22]. Large and giant breed dogs are particularly vulnerable to OA; however, all sizes and breeds can be affected due to age and being overweight [21,22,23]. This is why canine animal models were used in studying stem cell therapy for the treatment of OA. 

Dog is a good model for bone and joint diseases, since these are common in canines [24]. The genetic homology of healthy and abnormal tissues and conditions are more extensive than between humans and rodents. There is a close analogy between OA in humans and dogs [25]. Nonetheless, OA progression in humans is quite slow and it may occur over 15–30 years. Therefore, it is quite difficult to find an animal model that mirrors the human OA rate of progression [26]. The large animal models are undoubtedly better than smaller animal models for extrapolating results that may be useful in human medicine.

The prevalence of OA shows that musculoskeletal and joint diseases are age-related, and global statistics have shown that these pathological changes are a major health problem and financial burden for health and social welfare systems globally [4]. Osteoarthritis is one of the over 200 forms of arthritis known to exist; however, OA is the most prevalent form of the disease. The problem continues to grow not only due to the increase in the human lifespan, but also due to environmental changes and the adverse impact of a sedentary lifestyle coupled with a poor diet. In the USA, 10% of men and 13% of women over 60 years of age currently suffer from osteoarthritis of the knee. In 2030, the projected estimate is that 67 million Americans will be suffering from arthritis [26]. Worldwide, 10% of men and 18% of women over 60 years of age suffer from OA of the knee [2,3], and it has been estimated that OA affects 250 million people throughout the world [26]. More women than men suffer from OA, and the increase in the prevalence of OA in women after 50 has been linked to a decrease in estrogen levels [7]. Likewise, OA is a large-scale problem for veterinary medicine. More than 20%, which is 10 to 12 million dogs in the United States, are currently afflicted. In fact, osteoarthritis is the most common cause of chronic pain in dogs [18,21,23], which is another reason why canine models were used to study OA for human applications. 

Based on the results of studies conducted with the use of animal models as well as patients, there is a strong suggestion that a cascade of factors are involved in the pathological mechanism of OA [17,27]. This review will focus on the factors contributing to OA, as well as the mechanisms underlying it, and compare traditional treatments with newer regenerative therapies, which mainly use stem cell technology. These newer regenerative techniques have applications in both human medicine as well as veterinary medicine, but this review will focus mainly upon human treatment options. 

## 3. Stem Cells

Stem cells are precursor cells. The cells have the ability to self-renew, can stay in an undifferentiated state, show high plasticity, can transdifferentiate, and have quite a long life span. There are two pathways through which these cells can divide: (1) symmetric division—the daughter cell is identical to the antecessor and both cells remain as undifferentiated stem cells, or (2) asymmetric division—the daughter cell has limited developmental potential, which is the way stem cells differentiate and specialize into lineages [28]. These cells are unspecialized but can give rise to other specialized cell types [29]. There are two possible origins of stem cells. They may either be embryonic (ESCs), coming from a very early embryo or blastocyst, or they may be postnatal/adult stem cells (ASCs), which are undifferentiated, capable of self-renewal, and responsible for adult tissue regeneration [28,29].

There are various types of adult stem cells. There are hemopoietic stem cells (HSCs) that give rise to all blood cell components, including neutrophils, lymphocytes, natural killer cells, dendritic cells, macrophages, and monocytes. These HSCs are of mesodermal origin, are derived from bone marrow, and generate all blood cell types [28]. There are also mesenchymal stem cells (MSCs) that give rise to osteoblasts, chondrocytes, adipocytes, and the reticular stroma. Furthermore, there are neural stem cells, skin stem cells, and retinal stem cells, as well as peripheral blood stem cells (PBCSs), which show similarities to embryonal stem cells, are more immature than bone marrow mesenchymal stem cells (BM-MSCs), have proliferative potential, have an ability for multilineage differentiation, and have a trophic ability [30,31,32].

## 4. Mesenchymal Stem Cells (MSCs)

The most useful stem cell for tissue engineering and implantation for the treatment of OA are mesenchymal stem cells (MSCs). These are usually restricted to forming only mesodermal specific cell types (adipocytes, osteoblasts, myocytes, and chondrocytes), but several are able to differentiate into other cell varieties. The trophic effects of MSCs include the secretion of bioactive molecules that are anti-apoptotic, immunomodulatory, angiogenic, ant-scarring, and/or chemoattractant [32,33]. Stem cells in adults reside in niches, which are limited and have a specialized microenvironment. These cells have a physical anchoring site with a set of factors that control the cell number, activation, proliferation, self-renewal, or lineage differentiation. The microenvironment of the niche, with all its factors and signaling modulators, maintain homeostatic regulation of the stem cells by the up- or downregulation of the signaling pathways. In adults, the in situ source of bona fide MSCs has been identified in a perivascular location near pericytes and the tunica adventitia. These cells collectively are termed as perivascular stem cells (PSCs) [32,33]. MSCs are derived from perivascular cells and pericytes, and therefore could be derived from any vascularized tissue [34].

The paracrine effects of MSCs can be divided into three types: trophic, immunomodulatory, or chemoattractant. The trophic effects mainly stimulate neighboring parenchymal cells. These include the inhibition of apoptosis, and the support of regeneration, stimulation, maintenance, proliferation, and differentiation of tissue specific progenitors [32]. The immunomodulatory aspects may include an immunosuppressive effect, and immunoactivity mediation by direct cell–cell contact and by the secretion of bioactive molecules. The cells involved in interactions may include dendritic cells, B cells, T cells—including T regulatory cells and T helper cells—and killer cells. MSCs also secrete a variety of chemoattractant molecules. These target cells such as monocytes, eosinophils, neutrophils, memory and naïve T cells, B cells, natural killer cells, dendritic cells, and endothelial cell progenitors. These are the chemoattractant effects of MSCs [33].

Although MSCs can be found in various niches, they have many functional similarities. MSCs derived from various sources display different toll-like receptors (TLRs), which have functional properties, and respond to stimulation by TLRs agonists. TLRs are transmembrane proteins which play critical roles in the immune system by mediating inflammatory responses, primarily through the binding of ligands. MSCs are not spontaneously immunosuppressive, and the presence of inflammatory mediators may be essential for MSC-mediated immunosuppression and modulation of the functional properties. Results have shown that LPS-activated human WJ-MSCs (which mimic inflammation) express more TLR4 after 72 h when compared to non-activated cells; however, fetal tissue-derived MSCs seem to not be as sensitive to the LPS engagement as MSCs derived from adult tissues [35]. Therefore, the mechanism seems to be that TRLs modulate MSCs through MMPs (matrix proteinases).

## 5. Causes of Osteoarthritis

Unfortunately, chondrocytes may over-produce matrix-degenerating enzymes, such as matrix metalloproteinase 13 (MMP-13) [2,36]. While MMP-13 is needed for the healthy maintenance of articular cartilage, its overproduction can promote OA. In the osteoarthritic joint, there is a great mobilization of macrophages, and this consequently produces cytokines. The two major pro-inflammatory cytokines that have an impact on the progression of cartilage breakdown are IL-1β and TNF-α, which work by promoting catabolic and degradative processes. Experiments conducted on mice have suggested that a decrease in the TGF-β level (produced by synovial macrophages) induced osteophyte formation [17,36]. 

There are catabolic and pro-inflammatory mediators in OA, such as cytokines and nitric oxide, which play an important role in triggering the pathophysiology of OA by instigating the formation of free radicals (reactive oxygen species). The overproduction of cytokines triggers inflammatory stress that is responsible for degenerative and inflammatory tissue damage. Another type of mechanism is a destructive process activated by reactive oxygen species, which involves the induction of chondrocyte apoptosis [9]. For example, prostaglandin E_2_ (PGE_2_), which is produced by the inflamed synovium, leads to increased homeostatic imbalance (cartilage matrix degeneration, and regeneration), and overproduction of the proteolytic enzymes (which leads to cartilage breakdown) [4,11]. Moreover, the activation of TLRs leads to the activation of catabolic pathways in chondrocytes, and it was found that TLR-2 and TLR-4 were upregulated in OA [17]. The physiological microenvironment of the degenerated joint is likely to be hypoxic, acidic, deprived of nutrients, and exposed to higher-than-normal concentrations of pro-inflammatory cytokines and reactive oxygen species. All those conditions create an extremely difficult environment for effective regenerative therapy [4,36]. Table 2 summarizes the possible causes of osteoarthritis.

Multipotent mesenchymal progenitor cells (MSCs such as CD105^+^ and CD166^+^ progenitor cells isolated from cartilage) are present in human adult articular cartilage. It has been shown that the number of progenitor cells was higher in OA cartilage than in non-osteoarthritic joints [37]. However, some authors have suggested that the synovial fluid of a healthy joint does not contain MSCs [38]. A significantly greater number of MSCs in the OA joint may suggest that regenerative cells are attracted to the disease site. Nonetheless, the quality of cells is significantly reduced in advanced stages of OA [6,10]. In the synovial fluid of patients with articular cartilage degeneration and OA, higher levels of MSCs have been reported. Presumably, the synovial fluid in the OA joint might inhibit chondrogenic differentiation of the progenitors that are present [6,16]. 

## 6. Traditional Therapy and Models of OA

There is no effective therapy against the progression of OA. Currently, pain management, activity modification, and weight loss are prescribed in the early stages, but in the advanced stages there are very few options available. There are a few alternatives to help with moderate OA such as high tibial osteotomy of the knee joint, for example, in order to attempt to realign that particular joint. However, the patient must not be in too advanced of a stage of the disease [39]. Another traditional therapy has been hyaluronic acid; however, artificial hyaluronic acid can only provide temporary pain relief [40]. Joint replacement is generally the therapeutic procedure employed [41]. Animal models of OA of the knee have included horse, sheep, rat, mouse, rabbit, and guinea pig [41], as well as a caprine model (goats) [26] and dogs [42,43]. The large-animal models have had the most advantage in modelling the human progression of the disease, as compared to small-animal models, since they have a larger body, longer life, long-term follow-up, and are a better simulation of human pathology [44]. 

The main aim of traditional pharmacotherapy for OA is pain relief or reduction. Commonly used pharmacotherapies are acetaminophen, non-steroidal anti-inflammatory drugs, and opioid analgesics (tramadol). Intra-articular injections of corticosteroids are also applied; however, these treatments do not inhibit the decay process and adverse events are frequently noticed with prolonged use of these pharmacotherapies [2]. The prolonged administration of drugs is an inherent problem in most chronic diseases and is associated with possible gastrointestinal, renal, and hepatic adverse events [18]. The traditional multi-modal therapy of inflammation and pain reduction includes long-term cyclo-oxygenase-inhibiting non-steroidal anti-inflammatory drug (NSAID) therapy, physical therapy, diet, weight management, and dietary supplements. NSAIDs are a non-curative treatment. Moreover, there are suggestions that often pain relief is not complete [18,45]. 

During the early stages of OA treatment, physical therapy is involved, along with weight loss, body balance improvement, training in the reduction in mechanical stress, and pain reduction. Nutritional supplementation for joint support is commonly added to the diet. The most popular are glucosamine, chondroitin, and omega-3 fatty acids [21,23]. For advanced OA, total joint replacement is performed [2]. Total knee replacement is extremely expensive, employs a high amount of effort, and is not always successful [1,20].

The greatest problem with traditional OA treatment is that it does not stop the disease, but only focuses on damage reduction. In order to cure joint tissues, a new effective treatment is still being sought after. New medications have been targeted toward chondrogenesis induction, osteogenesis inhibition, matrix degradation inhibition, apoptosis inhibition, and anti-inflammatory cytokine effectiveness. There is hope that preclinical and clinical studies may help to manage these problems more effectively [2,5].

## 7. Regenerative Medicine

The most important and most difficult task of cartilage tissue engineering is creating a functional substitute for native cartilage [5,34]. In 1993, Langer and Vacanti defined tissue engineering as accentuating the interdisciplinary character to restore, maintain, or improve tissue function [3]. Regenerative therapy/cell therapy, especially with the use of stem cell technology, may one day fulfil the requirements of delaying OA progression and joint tissue repair [2,34]. In 1968 in the United States, the first successful allogenic stem cell graft in humans using donor bone marrow was undertaken [31]. This was perhaps the first step in using mesenchymal stem cell technology. In order to make the use of MSCs more practical in the future, much of the procedures would need to be standardized and less experimental. The basis for routine clinical MSCs applications would include standardized methods of isolation, characterization, and differentiation, as well as biocompatible scaffolds. It would also need to establish safety and efficiency levels [29], and standardized treatment protocols, guidelines, and dosing [31,36].

Chondrocyte implantations for cartilage regeneration has quite a long history, dating back to 1994 [46]. The clinical use in human patients actually began in 1987 [4]; however, since OA has such complex degenerative joint changes in different age groups of patients, the therapy was not fully effective. Mesenchymal stem cells are preferred since they may be collected from different tissues, are actively immunosuppressive, have a capacity for chondro-differentiation, and have a high proliferation potential [2,14]. Bone marrow and adipose derived MSCs have been most commonly used for OA treatment and repair. The disadvantage of autologous chondrocytes as regenerative cells is that they have a limited capacity to proliferate [18,47]. Autologous chondrocyte implantation was the first cell-based surgical strategy employed [6], but it is unfortunately limited to younger patients (<40 years) and, in this criterion, it is not suitable for the majority of patients with OA [46]. Monolayer-cultured chondrocytes tend to dedifferentiate, which means that they lose their characteristic phenotype and synthesize type I collagen (characteristically fibrocartilage) rather than type II collagen (characteristically hyaline cartilage). In this way, chondrocyte expansion is more complicated (vagarious) than MSCs, which are very stable and do not suffer the dedifferentiation process [19]. 

## 8. Mesenchymal Stem Cells and the Treatment of OA

As previously mentioned, but worth reiterating, there are two types of cells used for cartilage engineering: (1) chondrocytes, which were the first to be used and are obtained by the isolation and amplification of autologous chondrocytes unless the monolayer cultured chondrocytes rapidly lose their phenotype, and (2) mesenchymal stem cells (adult MSCs from different sources). Adult MSCs are of major interest in tissue engineering. MSCs that have already been applied have been sourced from bone marrow (most popular source), adipose tissue, muscles, periosteum, perichondrium, synovium [3,33], umbilical cord blood, as well as muscle and peripheral blood [1,48]. What should be considered is which type is the most suitable stem cell population for cartilage repair based on availability, effort of preparation, and chondrogenic potential. Furthermore, fibroblasts and genetically modified cells have been considered, but there is not much published research on the use of these cell types [1,3]. 

MSCs seem to provide some important advantages over chondrocytes when considering the treatment of degenerative joint diseases. They are easier to culture, more rapidly proliferate, and can specialize to become all tissues within the joint. Moreover, the paracrine activity seems to be most beneficial in treating OA conditions. The anti-inflammatory and immunomodulatory properties of MSCs play a pivotal role in orchestrating the reparative response of damaged joint tissues [36,41,49]. Mesenchymal stem cells (MSCs) interact with immune cells and are responsible for the modulation of a number of effector functions, immunomodulatory properties, migratory abilities, the induction of peripheral tolerance, inhibiting the release of pro-inflammatory cytokines, and the promotion of tissue repair. 

The advantage of using MSCs for treating OA include their capacity to differentiate into chondrocytes and their potential to prevent chondrocyte apoptosis and to prevent the overall process of degeneration (through a paracrine effect) [37]. They also modulate the activity of the immune system (via an immunosuppressive function), secrete cytokines and chemokines, suppress T cell proliferation, and inhibit the respiratory burst in neutrophils. The environment is responsible for modulating the balance between the pro-inflammatory and anti-inflammatory properties of MSCs [49]. Many pre-clinical and clinical trials have employed MSCs for OA management. The most important issues are the accuracy of evaluating the processes of disease progression, and the evaluation of cartilage regeneration. For quite some time, MSCs were regarded to be ‘immune privileged’, meaning that they are hypoimmunogenic. However, recent studies have suggested that MSCs may not be ‘immune privileged’, but “immune evasive”. If MSCs are only “immune evasive” rather than “immune privileged”, it could limit the long-term prospects of allogenic MSC transplantation, because eventually the immune system may notice these cells as foreign. However, more research needs to be done to clarify this issue [4,33,50]. In the meantime, the lack of an adverse immune response due to allogenic MSC administration is still a great advantage. 

The reason for allogenic MSC transplantation, rather than an autograft, is that there is a suspicion that OA patients, especially in advanced stages, are not the best donors of MSCs for their own treatment. Some authors have suggested that OA patients may have systemic depletion and derangement of MSCs. Cell differentiation and proliferation capacity may be too low to make a positive difference in the rebuilding of joint homeostasis. This negative impact on MSC dysfunction seems to be greater in bone marrow-derived cells than in MSCs derived from adipose tissue [41]. Filardo et al. identified 72 preclinical and 18 clinical studies with MSC usage for cartilage lesion treatment [51]. Vinatier and Guicheux reported 58 clinical studies involving MSCs for OA referenced at ClinicalTrials.gov [3]. There has been a growing trend of interest in cell therapies for cartilage regeneration in the last decade, as confirmed by the studies mentioned above. This mostly has to do with bone marrow-, adipose-, and synovial-derived MSCs, which have been used for treatment, with BM-MSCs used in the majority of cases [34,35,36].

The most important impact of MSCs on the regeneration of OA joints is the paracrine stimulation of the local microenvironment. MSCs have been shown to stimulate tissue regeneration via mesenchymal stem cell-derived paracrine signals [41]. However, the exact mechanisms of these processes are still being studied. Even the amount of MSCs to be used is still debated for the most part. For example, in the literature on canine MSCs, there is still a lack of information concerning the ideal cell dosage, systemically or locally applied, and the best cell source for each specific treatment is still debated. In human medicine, there is a range from 1 to 5 million cells/kg administrated, but it is not exactly clear how many MSCs are required to promote paracrine stimulation. In various species, cells from different sources differ in their specific properties, which suggests that cells should be carefully selected for the characteristics of a specific disease. For example, bone marrow-derived mesenchymal stem cells (BM-MSC) should be differentiated in vitro and should be expressing the correct markers for a chondrocyte, osteocyte, etc., depending on which properties are required [31,32,33,34,35,52,53]. 

Potential sources of MSCs for cartilage repair have been proposed, and they include bone marrow, adipose tissue, synovium, and Wharton’s jelly/umbilical cord, as previously mentioned. The advantages of these sources of MSC derivation are ease of harvesting, high proliferation rates, hypo-immunogenic properties, and non-tumorigenic abilities. The most important aspect for cartilage repair is the availability and chondrogenic differentiation potential [4,33].

In 2002, the first publication with regards to OA treatment with autologous, bone marrow-derived MSCs appeared. A significant improvement was reported, because between 5 and 135 months of follow-up, no tumors or infections were observed [49]. In 2003, the first large animal model of OA, caprine, was used for studies of MSC transplantation. Twenty weeks after injection, the reduction in OA symptoms was noticeable with less subchondral sclerosis, a remodeling of the articular cartilage, and fewer osteophytes [49]. The results of MSC administration in animal models of OA varied due to the animal model and/or injury, treatment timing, type of MSCs, culture method, and dose [54,55]. However, much of this gave a starting point for human clinical trials, and although the results have been encouraging, a very large number of MSCs are currently required. This is because the MSCs tend to migrate away from where they are required after intra-articular injection. This has led to some current research on modifying the MSCs in order to better target them to the affected area [33]. These techniques are still in the early stages of investigation but may include cell surface modifications or magnetic-assisted tissue targeting. Unfortunately, these techniques are not currently available clinically. 

The restoration of a fully functional, structurally, and mechanically, articular cartilage surface has not been achieved to date [1], which demonstrates how challenging the repair treatment of cartilage really is. There are potential risk factors of mesenchymal cellular therapy as well. They include the differentiation into undesired cell types, ectopic tissue formation, the transformation into a tumor, a potential immune response in the case of allogenic transplantation, unpredicted adverse events, MSC-mediated endochondral ossification, and scar tissue formation [49,50,51,52,53,54,55]. Although some of these risks, such as MSC malignancy are rare, they still should seriously be taken into account due to their resistance to chemo- and radiation-therapy [56]. They also tend to frequently metastasize in their advanced stages. Meanwhile, the unpredicted adverse events vary widely, but most often include transient fever, adverse reactions at the administration site, fatigue, constipation, and insomnia [56,57]. Table 3 summarizes the potential risk factors of mesenchymal stem cell therapy.

## 9. Conclusions

In conclusion, untreated osteoarthritis will not heal spontaneously, and current standard treatments are very limited due to the lack of vascularization in the cartilage tissue. Therefore, stem cell therapy seems to be the most promising for the regeneration of joint tissue, especially in the middle to late stages of the disease. Of the various stem cell types, mesenchymal stem cells are the most promising since they are relatively easy to harvest, proliferate very well, do not cause tumor formation, and are very well tolerated by the immune system. Hopefully, in the near future, it will be relatively routine to treat patients with this technology, since it has progressed relatively rapidly from animal models to chondrocyte transplantation, and then to our current state of bone marrow-derived MSC therapy. 

## Figures and Tables

**Table 1:** Summary of the Characteristics of the Four Main Zones of Articular Cartilage.

Zone	Characteristics of Collagen	Characteristics of Chondrocytes	Main functional Characteristic
Superficial (tangential) Zone	Primarily type II and IX collagen packed tightly and aligned parallel to articular surface	Chondrocytes mainly flattened	Protects the deeper layers from shear stress and is in contact with the synovial fluid
Middle Zone	Thicker fibrils arranged obliquely surrounded by a large number of hydrated proteoglycans	Chondrocytes are spherical and have a very low density	Resists compressive forces and functions as a bridge between the superficial and deep zones
Deep (Basal) Zone	Large diameter fibrils arranged radially and perpendicular to the articular surface with a large number of dehydrated proteoglycans	Chondrocytes are columnar and run parallel to the collagen fibers and perpendicular to the joint line	Highly resistant to compressive forces and is the last zone before the tidal mark
Calcified Zone	Fibrils arranged perpendicular to the articular surface with a large number of proteoglycans	Very few chondrocytes and most are hypertrophic	Greatest resistance to compressive forces and functions mainly to anchor the cartilage to the bone

**Table 2:** Summary of Possible Causes of Osteoarthritis.

Possible Cause	Specific Effect	Clinical Result	Reference Number
Matrix Degenerating Enzymes	Overproduction of matrix metalloproteinase 13 (MMP-13)	Enzymatic destruction of the extracellular matrix	Zhang et al. [2] Freitag et al. [36]
	Production of other disintegrin enzymes	Enzymatic destruction of the extracellular matrix	Zhang et al. [2] Freitag et al. [36]
Exaggerated Immune Response	Mobilization of macrophages	Catabolic processes	Haseeb and Haqqi [17]
	Cytokine production	Presence of IL-1β and TNF-α, which trigger catabolic and degradative processes	Haseeb and Haqqi [17] Djouad et al. [36]
	A decrease in the TGF-β level	Increased osteophyte formation	Haseeb and Haqqi [17] Djouad et al. [36]
Catabolic and Inflammatory Mediators	Overproduction of cytokines and nitric oxide	Creation of Reactive Oxygen Species (free radicals) that cause oxidative and inflammatory stress	Mortellaro [9]
	Overproduction of proteolytic enzymes	Leads to cartilage breakdown	Mobasheri et al. [4] Jiang et al. [11]
	The activation of toll like receptors (TLRs)	Upregulation of TLR-2 and TLR-4, which activates catabolic pathways in chondrocytes	Haseeb and Haqqi [17] Freitag et al. [36]

**Table 3:** Summary of the potential risk factors of mesenchymal stem cell therapy.

Potential Risk Factors of Stem Cell Therapy		Reference Number
Possible risk factors include:	Differentiation into undesired cell types	Mobasheri et al. [4] Huang et al. [33] Boehme et al. [54]
	Ectopic tissue formation	Huang et al. [33]
	Transformation into a tumor	Boehme et al. [54]
	Potential immune response with allogenic transplantation	Baranovskii et al. [55] Wang et al. [56]
	Msc-mediated endochondral ossification	Huang et al. [33] Boehme et al. [54]
	Scar tissue formation-fibrocartilage	Mobasheri et al. [4] Huang et al. [33] Le et al. [14]
	Unpredicted adverse events	Baranovskii et al. [55] Wang et al. [56]

## Data Availability

No new data were created or analyzed in this study. Data sharing is not applicable to this article.

## Abbreviations

ASC	Adult/postnatal stem cell
BM-MSC	Bone Marrow-MSC
ESC	Embryonic Stem Cell
HSC	Hemopoietic stem cell
IL	Interleukin
LPS	Lipopolysaccharide
MMP	Matrix proteinase
MSC	Mesenchymal stem cell
OA	Osteoarthritis
NSAID	Non-steroidal anti-inflammatory drugs
PBSC	Peripheral blood stem cell
PGE	Prostaglandin E
PSC	Perivascular stem cell
TGF	Transforming growth factor
TLR	Toll-like receptor
TNF	Tumor necrosis factor
